# Volumetric analysis of simulated bone defects: a comparative study using cone beam computed tomography and intraoral scanners

**DOI:** 10.1038/s41598-025-31257-x

**Published:** 2025-12-19

**Authors:** Esraa A. ElMekkawy, Yousria S. Gaweesh, Shaimaa M. Abu El Sadat, Lobna M. Elsaadawy

**Affiliations:** 1 Faculty of Dentistry, Alexandria University, Alexandria, Egypt; 2 Faculty of Dentistry, Ain Shams University, Cairo, Egypt

**Keywords:** CBCT segmentation, Intraoral scanner, Volumetric analysis, Osteolytic defects, Bone defect measurement, Anatomy, Diseases, Health care, Medical research

## Abstract

This in vitro study aimed to evaluate and compare the accuracy and reliability of cone-beam computed tomography (CBCT) and intraoral scanner (IOS) in measuring the volume of simulated bone defects. Additionally, it aimed to assess how the number of missing cortical bony walls in a bone defect affects the accuracy of volumetric measurements. Bovine rib blocks were used to create 42 simulated bone defects, which were then divided equally into two groups. One cortical plate was perforated in Group I, whereas both the buccal and lingual cortical plates were perforated in Group II. Defect volumes were assessed using CBCT and IOS. Every measurement was compared to a gold standard derived using Archimedes’ principle. Volumetric analysis revealed that the IOS measurements in both groups showed no statistically significant difference when compared to the gold standard. CBCT measurements in Group I also showed no significant difference from the gold standard. However, in Group II, CBCT volumetric measurements differed significantly from the gold standard (*p* = 0.001). Both CBCT and IOS demonstrated accuracy in measuring bone defects when only one cortical wall was perforated. However, when both cortical plates were lost, IOS showed superior accuracy compared to CBCT segmentation. IOS measurements had no significant deviation from the gold standard volume (*P* = 0.88), while CBCT measurements exhibited a significant deviation (*P* = 0.001). Therefore, IOS presents a reliable, radiation-free alternative for monitoring volumetric bone defects across different degrees of cortical bone loss.

## Introduction

In dentomaxillofacial radiology, the integration of CBCT has revolutionized imaging by providing comprehensive anatomical information with reduced radiation exposure^1^. Advancements in this technology have introduced CBCT segmentation, a computer-based technique essential for accurately evaluating maxillofacial bone defects and estimating defect volume. This quantitative assessment guides treatment planning in trauma, pathology, congenital anomalies, and surgical cases by determining graft requirements, selecting suitable techniques, and predicting reconstruction success. Moreover, volumetric analysis facilitates postoperative monitoring, graft integration assessment, and outcome comparison, ultimately enhancing both functional and aesthetic results^2^.

Osteolytic defects characterized by the partial or complete absence of one or more bony walls present significant challenges during CBCT segmentation and volumetric analysis. Defects with fewer intact cortical walls exhibit open geometries that lack clear, stable anatomical boundaries. Standard segmentation methods often rely on continuous bone surfaces to accurately define defect margins. Consequently, this complexity can lead to less precise volume estimates, making it harder to reliably quantify the extent of bone loss or lesion size, which is crucial for treatment planning and surgical decision-making^3^.

The use of IOS offers a non-invasive, radiation-free alternative for assessing bone defects, providing real-time, high-resolution 3D images that enhance clinical evaluation, patient comfort, and procedural efficiency. However, the loss of cortical walls can alter defect geometry, creating open and irregular shapes that lack stable reference surfaces, which may limit the ability of IOS to accurately delineate defect boundaries. Consequently, in cases of partially or completely missing walls, the precision of volumetric assessment and the reliability of measurements may be affected, influencing diagnostic and treatment decisions^4,5^.

While previous research has explored the use of CBCT and, to a lesser extent, IOS in assessing bone defect volumes, there remains a significant gap in understanding how the morphological characteristics of osteolytic defects, particularly the number of remaining bony walls, affect the accuracy of these imaging modalities^4,5^. Therefore, our study aims to evaluate and compare the accuracy and reliability of CBCT and IOS in measuring the volume of two simulated bone defects. These defects differ based on the number of destructed cortical walls: one type has one destructed cortical wall, while the other has two.

The null hypothesis of this study is that there is no significant difference in volumetric measurement accuracy between CBCT and IOS across both types of simulated osteolytic bone defects: those with one destructed cortical wall and those with two destructed cortical walls. This hypothesis assumes that morphological variations in cortical wall integrity do not influence the comparative performance of the two imaging modalities in volume estimation.

## Materials and methods

The study was conducted after obtaining approval from the Research Ethics Committee, Faculty of Dentistry, Alexandria University. (IRB No. 0010556- IORG 0008839) before any research-related activities.

### Sample size calculation

The minimal sample size was calculated based on a previous study that assessed the accuracy of different CBCT voxel sizes in evaluating various infra-bony defects (both linear and volumetric), as well as the accuracy of two different software systems in volumetric measurements of alveolar bone defects^6^. Based on the findings of Hatata et al. (2019)^6^ and anticipating an Intraclass Correlation Coefficient (ICC) of 90% (0.9) and k (number of measurements of each specimen) = 2, the corrected sample size (nc) is 21 specimens per group (number of groups = 2): (Total sample size 42 specimens) at significance level of 5% (α error accepted = 0.05), and statistical power (1 – β) of 80%. Any sample withdrawal from the study due to a processing error will be replaced to maintain the minimum sample size^7^.

### Preparation and sectioning of bovine rib specimens

**Fig. 1:**
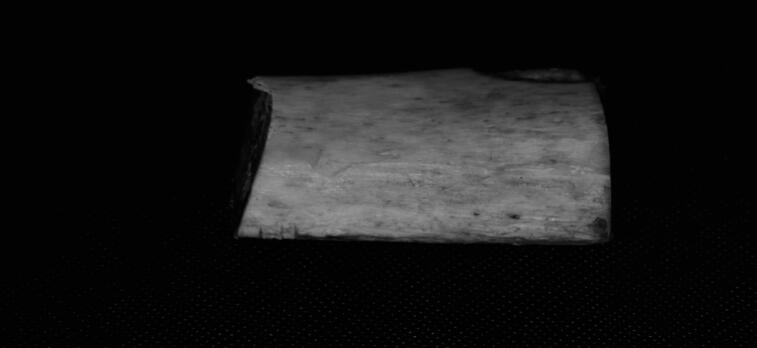
Prepared bovine rib block.

To simulate human alveolar bone, fresh bovine ribs were obtained from a local slaughterhouse. After precise removal of all overlying soft tissues, the rib ridges were flattened using a surgical bur to create a uniform bone surface. Then, the ribs were cut into forty-two small blocks, each with a uniform thickness of approximately 6–7 mm (Fig. 1).

### Sample grouping and defect preparation

Forty-two bovine bone blocks were randomly divided into two equal groups (Group I and Group II, *n* = 21 each). To minimize variability in cortical thickness and bone density, blocks obtained from the same rib were randomly assigned to the two experimental groups. Each block was labelled with a unique code indicating its rib of origin by an investigator, who was not involved in the randomization process. Randomization was manually performed using a simple random allocation method by an operator who was blinded to the labelling and the characteristics of the blocks. Two identical opaque containers labeled “Group I” and “Group II” were prepared, and for each rib, the corresponding blocks were placed in a separate tray and drawn blindly by the independent operator until half were assigned to Group I and the remaining half to Group II.

#### Group I

**Fig. 2:**
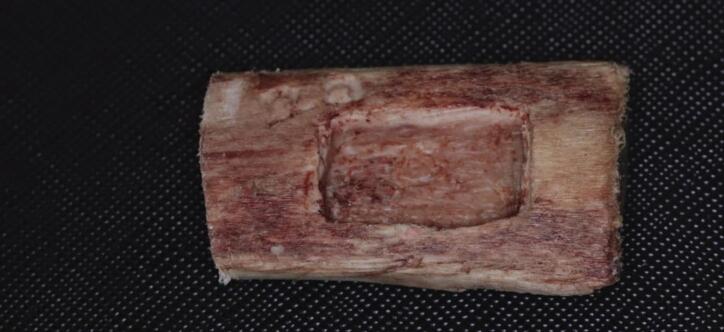
Bovine rib block prepared with a Group I defect.

Osteolytic defects were created by perforating one cortical plate, while the opposite plate remained intact (Fig. 2).

#### Group II

**Fig. 3:**
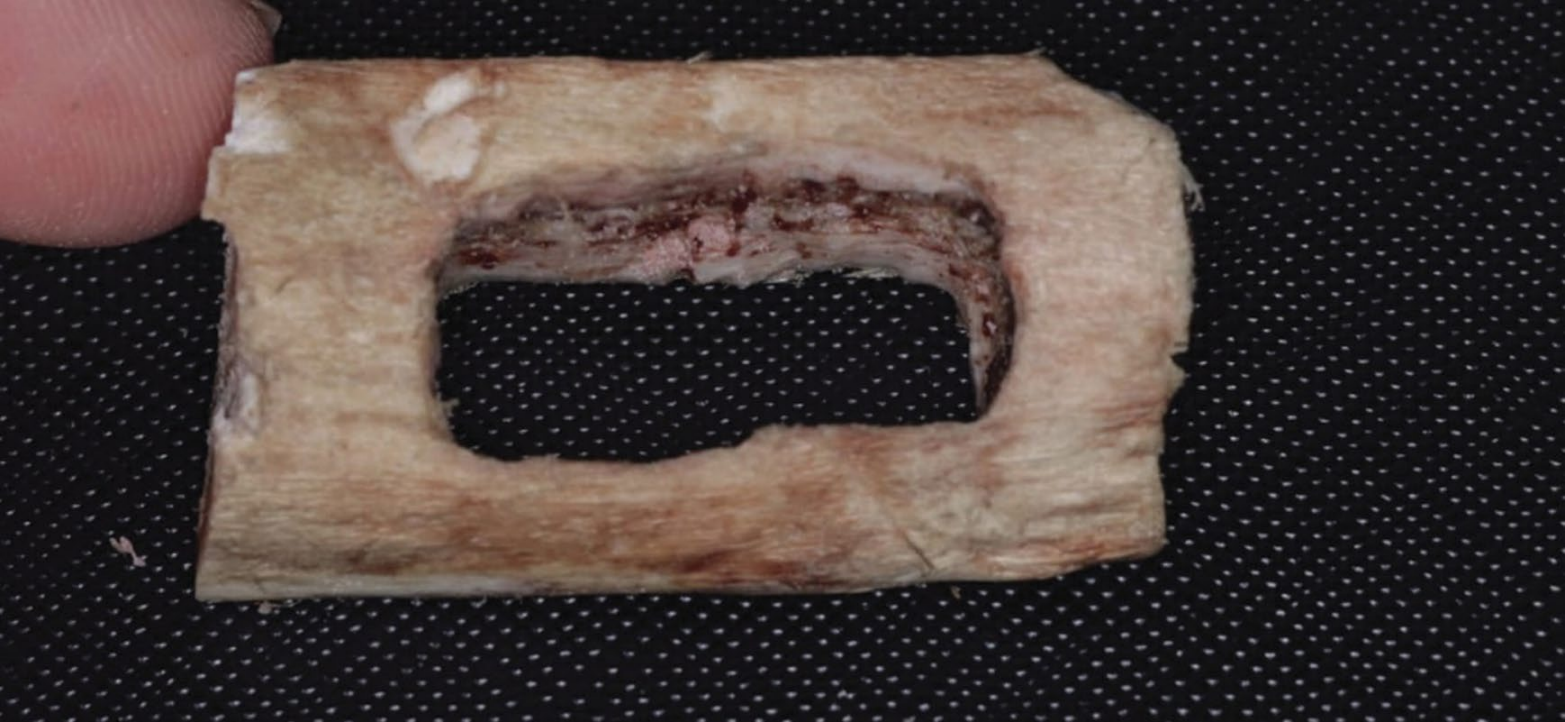
Bovine rib block prepared with a Group II defect.

A different type of osteolytic defect was simulated by creating through-and-through defects, involving the destruction of both cortical plates (Fig. 3).

Defects were created using surgical burs, with standardized dimensions of 20 mm in length, 10 mm in width, and 6 mm in depth. An endodontic ruler was used to ensure consistent length and width, while a periodontal probe was employed to verify the depth. To simulate soft tissue, each bone block in both groups was wrapped with a wax sheet (Cavex, Netherlands).

### Physical volume measurements (gold standard)

**Fig. 4:**
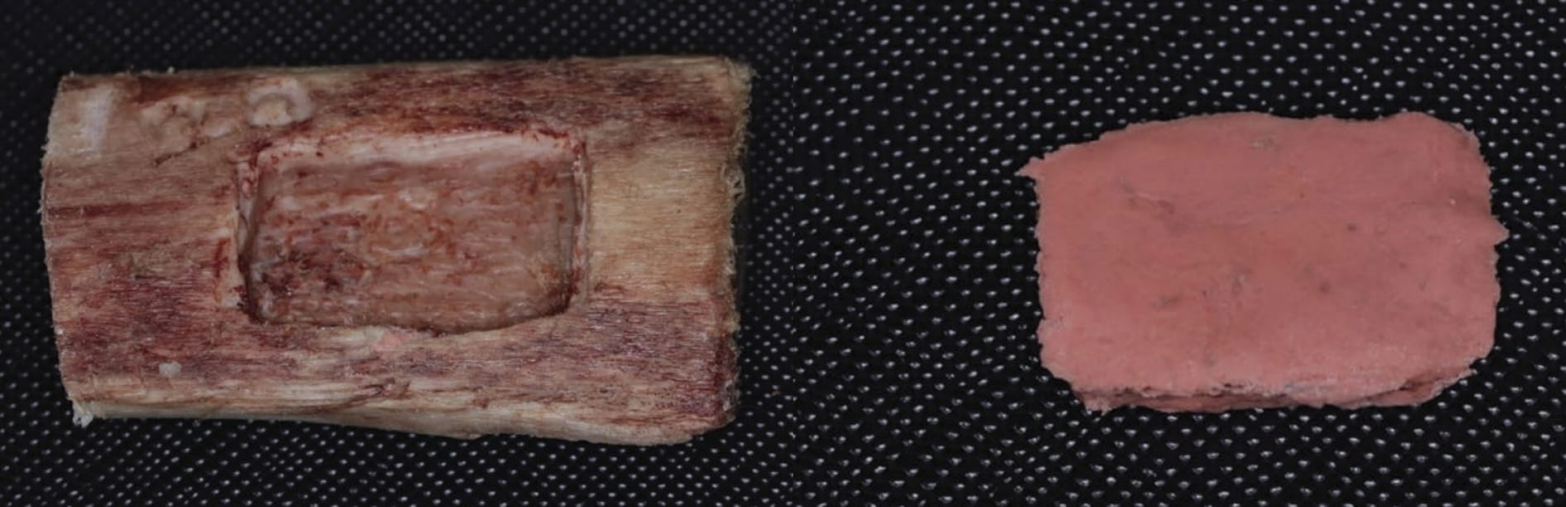
The replica was taken out from the defect to be weighed.

To establish a gold standard for volume measurements, a silicon-based impression material (Silaxil, Lascod, Ultimate Dental Supply Pty Ltd) was employed to create precise replicas of each simulated bone defect. The process began with the application of a fine layer of Vaseline to the inner surface of each bone defect to facilitate easy separation of the impression material after polymerization. The silicon-based impression material was then carefully filled into each bone defect to ensure a tight fit with the defect’s borders. After polymerization, any excess material was meticulously trimmed to maintain confinement within the defect boundaries. Each replica was subsequently removed from its respective defect (Fig. 4). The mass of every replica was measured using an analytical balance (RADWAG Wagi Elektroniczne, Poland), and the volume of each replica and consequently the corresponding bone defect was calculated using Archimedes’ principle by dividing the mass of the replica by the specific density of the Silaxil silicon impression material.

### CBCT scanning

The Blocks were scanned with an i-CAT CBCT machine (Imaging Sciences International, Hatfield, PA, USA). Acquisition parameters were as follows: 120 kVp, 18.54 mAs, 8.9 s scan time. Voxel size and field of view were set to be 0.25 mm voxel, and 16 × 4 FOV.

The relatively low mAs value, in combination with a limited field of view, was appropriate for scanning small specimens such as bovine rib blocks, reducing unnecessary radiation dose without compromising image quality.

### Digital segmentation and volumetric analysis using MIMICS software

Digital Imaging and Communication in Medicine (DICOM) data were processed for segmentation and volumetric measurement of the bone defects using Mimics software version 21.0 (Materialise NV, Leuven, Belgium). The DICOM data were first imported into Mimics, where intensity thresholds were adjusted to optimize visualization of both the bone structure and the created defects. A threshold range of -1024 to 1972 Hounsfield Units (HU) was applied to capture both the high-density bone and the lower-density defect regions effectively.

**Fig. 5:**
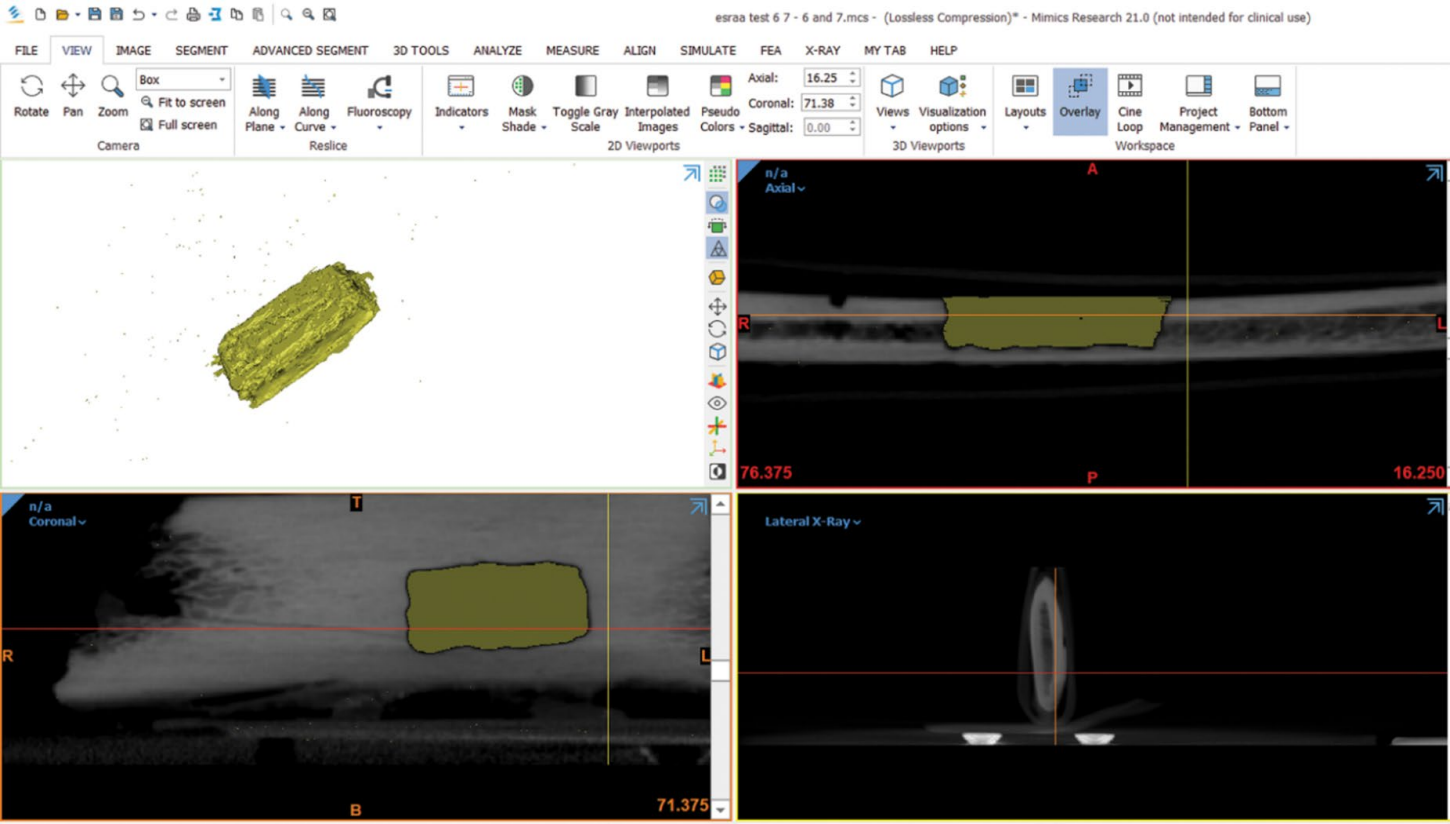
Manual segmentation of the defect (group (I)) and the resulting 3D volume.

**Fig. 6:**
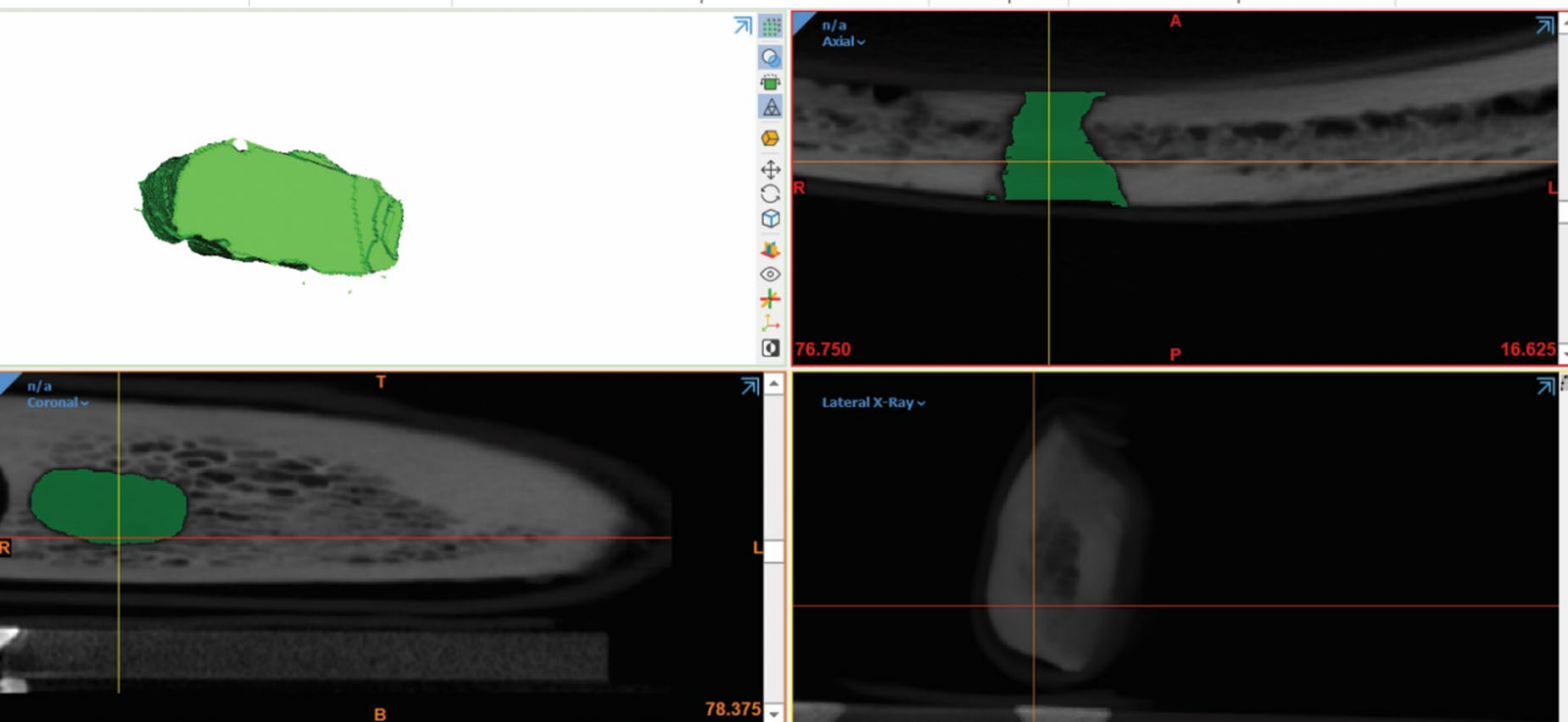
Manual segmentation of the defect (group (II)) and the resulting 3D volume.

Each defect was manually segmented by outlining its borders slice by slice on coronal sections, with the borders visually marked using color delineation. The segmentation on coronal slices was then verified and refined using the axial view to ensure anatomical accuracy and consistency across slices. Following segmentation, a 3D model of each defect was generated using the software’s 3D object reconstruction function. Finally, the volume of each defect was calculated using the “Properties” tool within the software. (Figures 5 and 6) illustrate the segmentation procedure and the final 3D volume of the defect on Mimics software.

All digital segmentations on Mimics software were performed by operators who were blinded to the gold-standard physical measurements obtained from the silicone replicas. The operators had access only to the CBCT DICOM data and were unaware of the corresponding Archimedes-based volume measurements to prevent measurement bias.

### Inter-observer reliability assessment

To ensure the reliability of the volume calculation process, a subset of 10 cases, representing 25% of the total cases was selected for repeated measurement by two independent investigators. This approach aimed to evaluate inter-observer reliability. Both examiners worked independently, without knowledge of each other’s results, to eliminate potential bias. They adhered to identical manual segmentation protocols and utilized laptops with uniform screen sizes (15.1 inches) to maintain consistency in their assessments.

### Optical scanning of silicone replicas and volumetric analysis

**Fig. 7:**
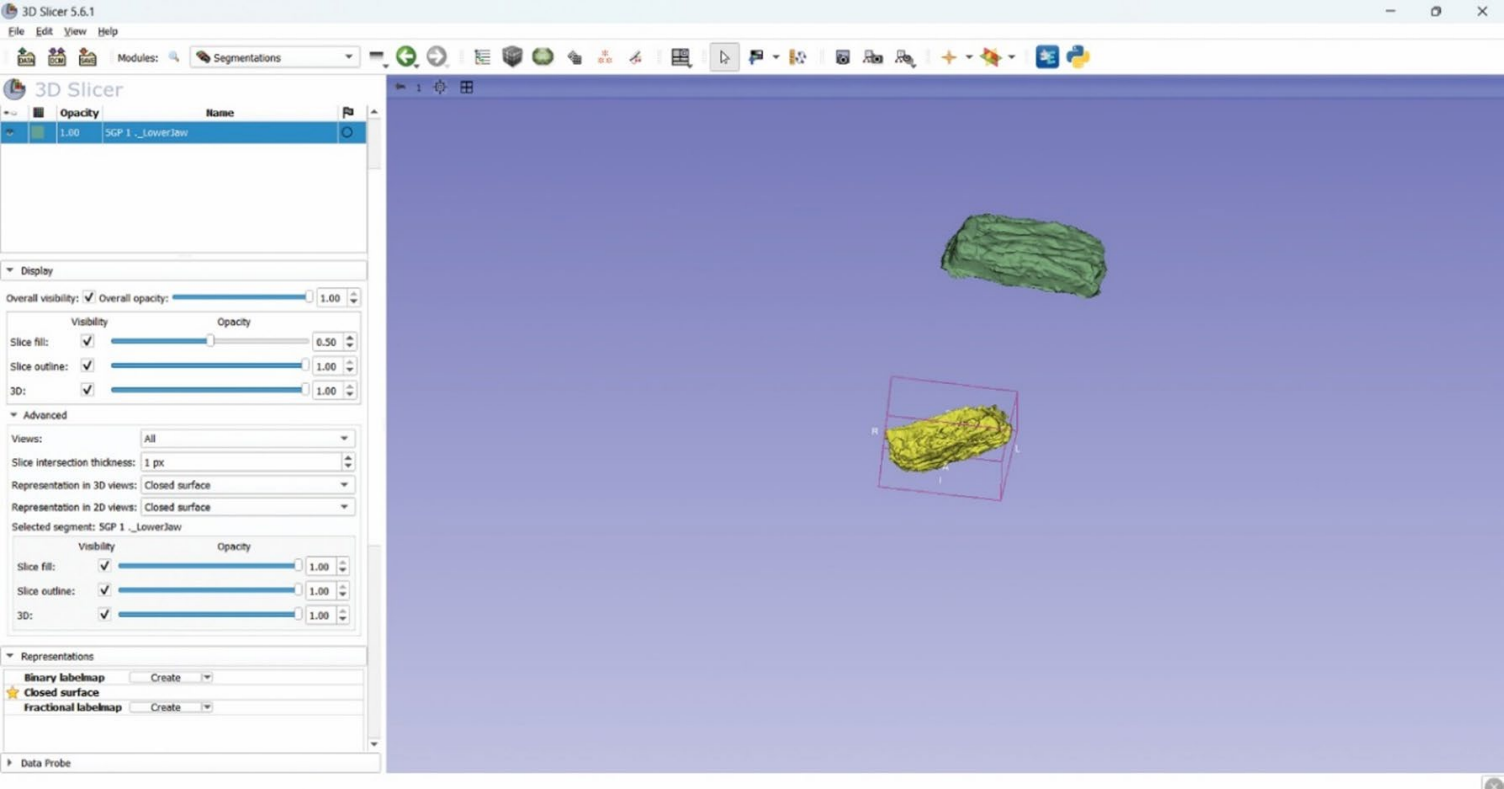
Virtual models obtained from IOS and imported into 3D slicer software for volume calculations.

The volume of the silicone impression replicas of the osteolytic defects was analyzed using a digital workflow. The silicone impression replicas were scanned using the Omnicam intraoral scanner (CEREC, Sirona, Bensheim, Germany), a high-resolution optical device designed for precise surface capture. The resulting digital impressions were exported in STL (Standard Tessellation Language) and imported into 3D Slicer software version 5.8.1 (Slicer Community, www.slicer.org), an open-source platform for biomedical image processing and analysis. Each replica scan was loaded as an individual segment for further evaluation (Fig. 7). The Segmentation Statistics tool within the software was used to automatically compute the volume of each segment.

To assess the accuracy of the CBCT and IOS-based volume measurements, the data obtained from these digital methods were compared to the reference volumes derived from theArchimedes principle, considered the gold standard. For each defect, the volume estimated using CBCT and IOS workflows was evaluated against the corresponding physical measurement. This comparison was performed separately for each defect group to analyze potential differences in accuracy between the two digital methods.

The mean time required for volume analysis was also recorded for each method. The CBCT-based workflow had an average segmentation time of 30 min per defect, while the IOS-based workflow required only 2 min per defect.

### Statistical analysis

SPSS version 23.0 for Windows (SPSS Inc., Chicago, USA) was used to analyze our data. The significance level was set at *p* < 0.05. The Kolmogrov-Smirnov and Shapiro-Wilk tests were used to ensure the quantitative data’s normality. All data showed a non-normal (non-parametric) distribution. Volumetric analysis of bone defects was described by median, interquartile range (IQR), mean and standard deviation (SD). The Wilcoxon-Signed rank test was used to evaluate the association between IOS and CBCT in comparison to the gold standard segmentation methods. The ICC was used to evaluate inter-/intra-examiner agreement level.

## Results

A descriptive analysis of statistical data (Volumetric analysis of bone defects described by median (IQR), mean and SD is shown in (Table 1).

**Table 1:** Descriptive volumetric analysis of bone defects using IOS, CBCT, and gold standard techniques (*N* = 42).

Group	Analysis methods	Median (IQR)	Mean ± SD
Group A (*n* = 21)	IOS	1018.69 (871.08, 1137.74)	1021.38 ± 184.09
	CBCT segmentation	1005.69 (798.20, 1130.81)	976.46 ± 219.70
	gold standard	1000.00 (902.94, 1108.82)	1012.25 ± 162.61
Group B (*n* = 21)	IOS	1578.12 (1395.33, 1785.37)	1649.92 ± 361.16
	CBCT segmentation	1807.86 (1400.35, 1897.36)	1,802.59 ± 417.68
	gold standard	1564.71 (1423.76, 1801.76)	1643.55 ± 344.36

*IQR* interquartile range, *SD* standard deviation.

### Volumetric analysis of group I

The Wilcoxon Signed-Rank Test revealed no statistically significant difference between the IOS measurements and the gold standard (*p* = 0.88). Likewise, CBCT segmentation measurements showed no significant difference from the gold standard (*p* = 0.26) (Table 2).

### Volumetric analysis of group II

There was no statistically significant difference between IOS measurements and the gold standard (*p* = 0.88). However, a statistically significant difference was observed between CBCT segmentation and the gold standard (*p* = 0.001) (Table 2).

**Table 2:** Association between the IOS, CBCT and gold standard techniques in the volumetric analysis of bone defects in group A and B (*N* = 42).

Group	Analysis methods	95% CI	*p* value
Group A (*n* = 21)	IOS and gold standard	(-17.52, 35.79)	0.88
	CBCT segmentation and gold standard	(-81.06, 9.48)	0.26
Group B (*n* = 21)	IOS and gold standard	(-23.88, 36.63)	0.88
	CBCT segmentation and gold standard	(-11.48, 196.89)	0.001*

Wilcoxon-Signed rank test.

*CI* confidence interval.

*Statistically significant at *p* < 0.05.

### Agreement between IOS and CBCT segmentation in comparison to the Archimedes method

ICC indicated excellent agreement between IOS and the gold standard (ICC = 0.97–0.99), as well as good agreement between CBCT and the gold standard (ICC = 0.89–0.92) (Table 3).

**Table 3:** Reliability between IOS and CBCT segmentation methods as compared to the gold standard in the volumetric analysis of bone defect in group I and II (*N* = 42).

Group	Analysis methods	ICC	95% CI	*p* value
Group A (*n* = 21)	IOS and gold standard	0.97	(0.93, 0.99)	< 0.001***
	CBCT segmentation and gold standard	0.92	(0.81, 0.97)	< 0.001***
Group B (*n* = 21)	IOS and gold standard	0.99	(0.98, 0.996)	< 0.001***
	CBCT segmentation and gold standard	0.89	(0.76, 0.96)	< 0.001***

ICC intraclass correlation coefficient, CI confidence interval.

***Statistically significant at *p* < 0.001.

### Interexaminer and intraexaminer reliability

The segmentation of the defects using MIMICS software revealed good inter-examiner agreement, with an ICC value of (0.87). Furthermore, the intraexaminer agreement was very good, with an ICC value of (0.92) (Table 4).

**Table 4:** Inter-observer and intra-observer reliability in performing volumetric analysis of bone defects using CBCT segmentation technique.

Reliability	ICC	95% CI	*p* value
Interobserver reliability	0.87	(0.11, 0.96)	0.01*
Intraobserver reliability	0.92	(0.23, 0.99)	< 0.001***

ICC intraclass correlation coefficient, CI confidence interval.

*Statistically significant at *p* < 0.01.

***Statistically significant at *p* < 0.001.

#### Bland altman plots

**Fig. 8:**
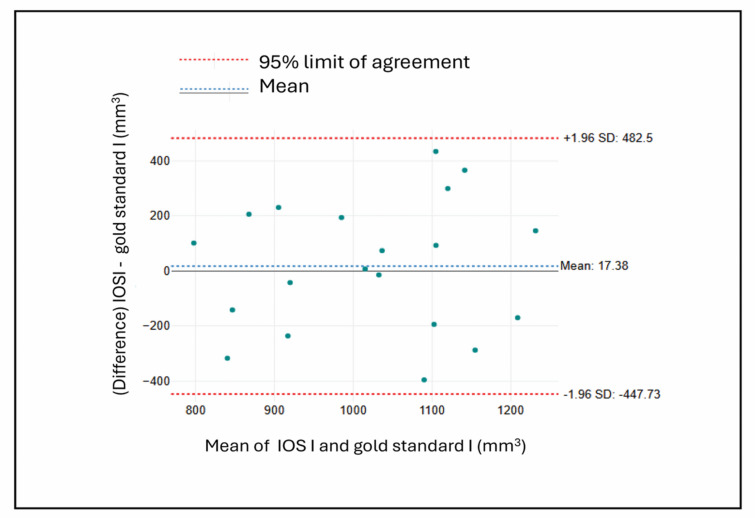
Bland-Altman plot comparing IOS and gold standard measurements in group I.

**Fig. 9:**
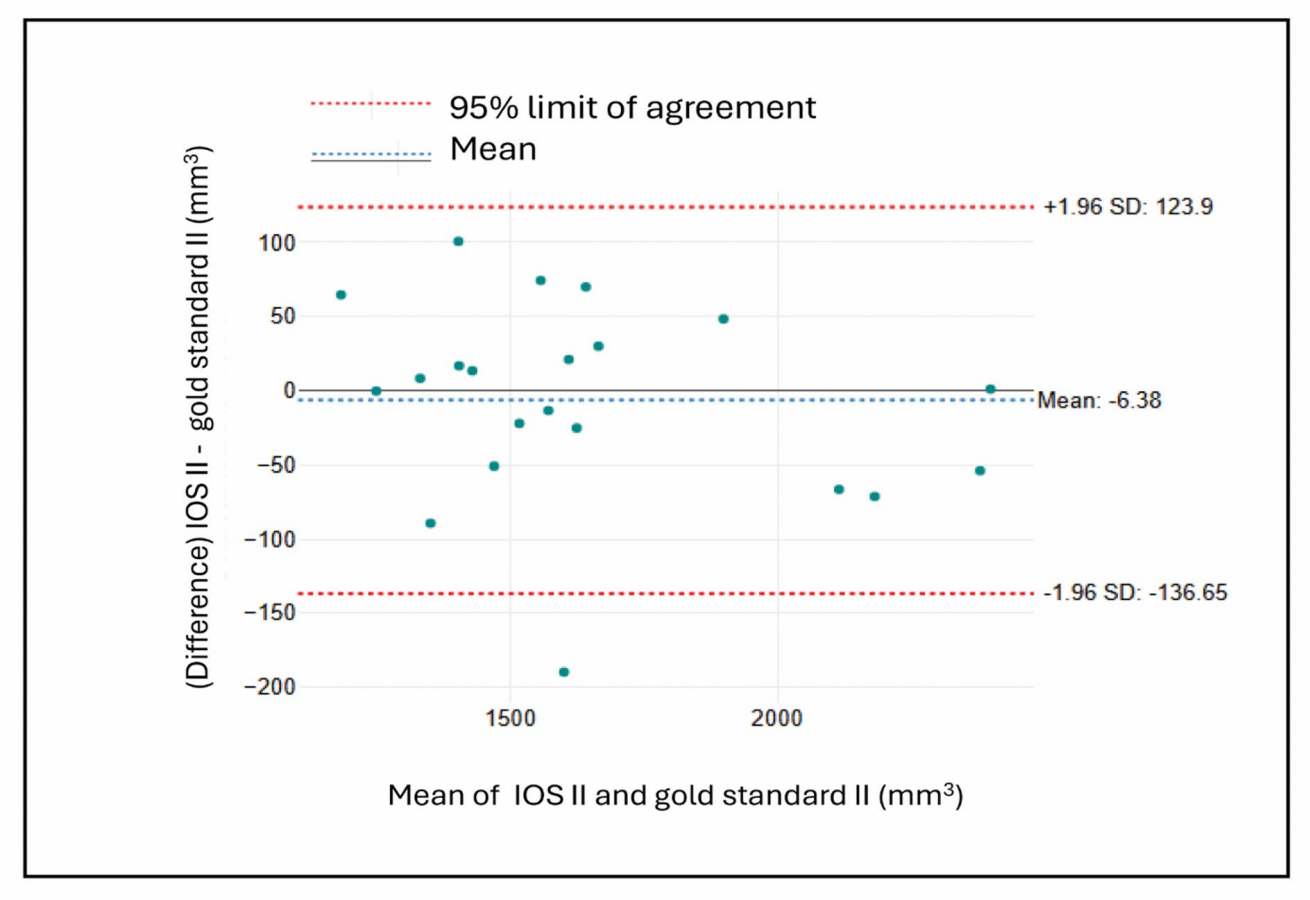
Bland-Altman plot comparing IOS and gold standard measurements in group II.

The four Bland-Altman plots offer visual assessments of agreement between each measurement method and the respective gold standard. The Bland-Altman plots for the IOS measurements (Figs. 8 and 9) demonstrate generally good agreement with the gold standard in both groups. Both plots show small mean differences (17.38mm^3^ for IOS I and − 6.38 mm³ for IOS II), indicating minimal systematic bias in each case.

**Fig. 10:**
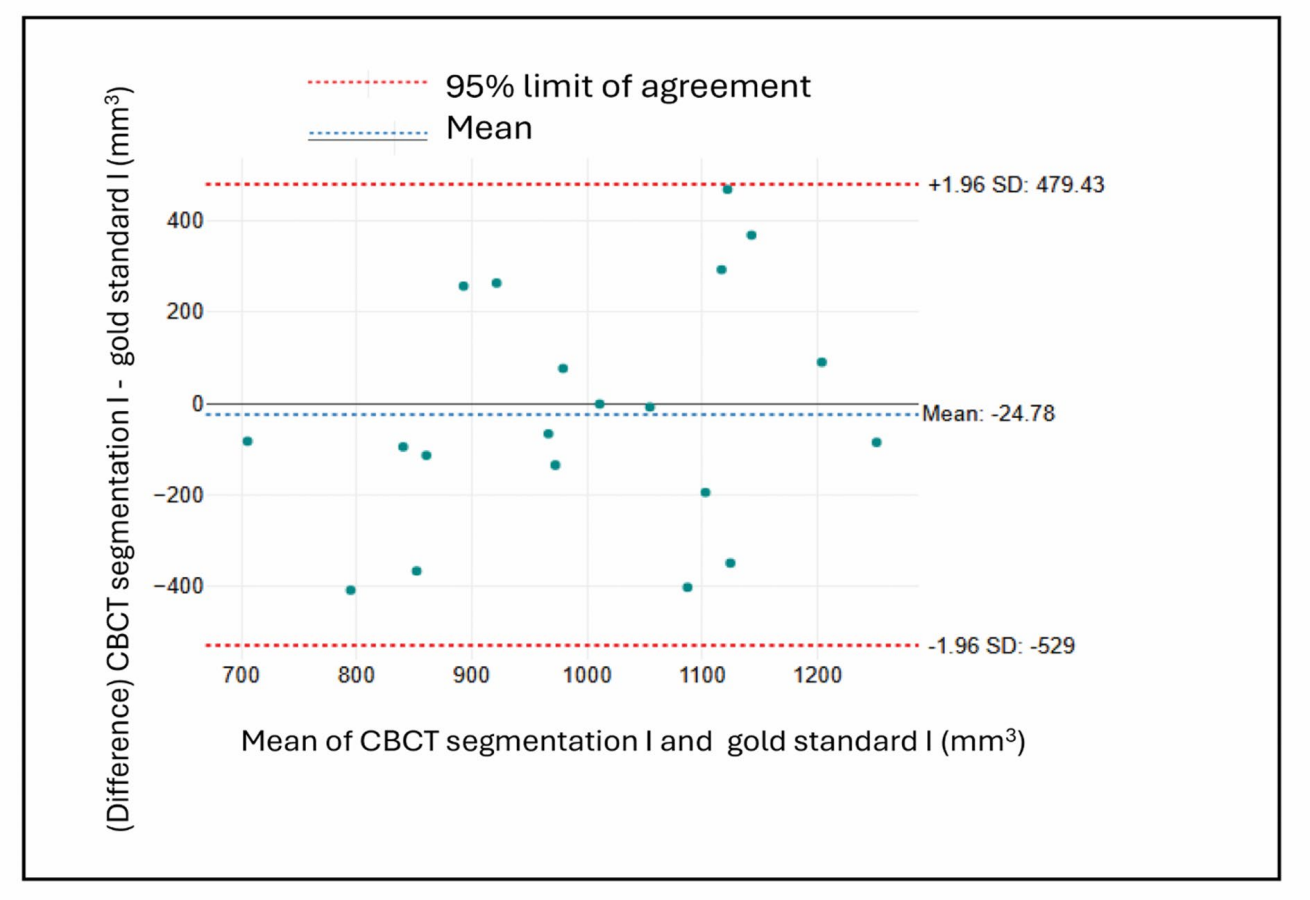
Bland-Altman plot comparing CBCT and gold standard measurements in group I.

**Fig. 11:**
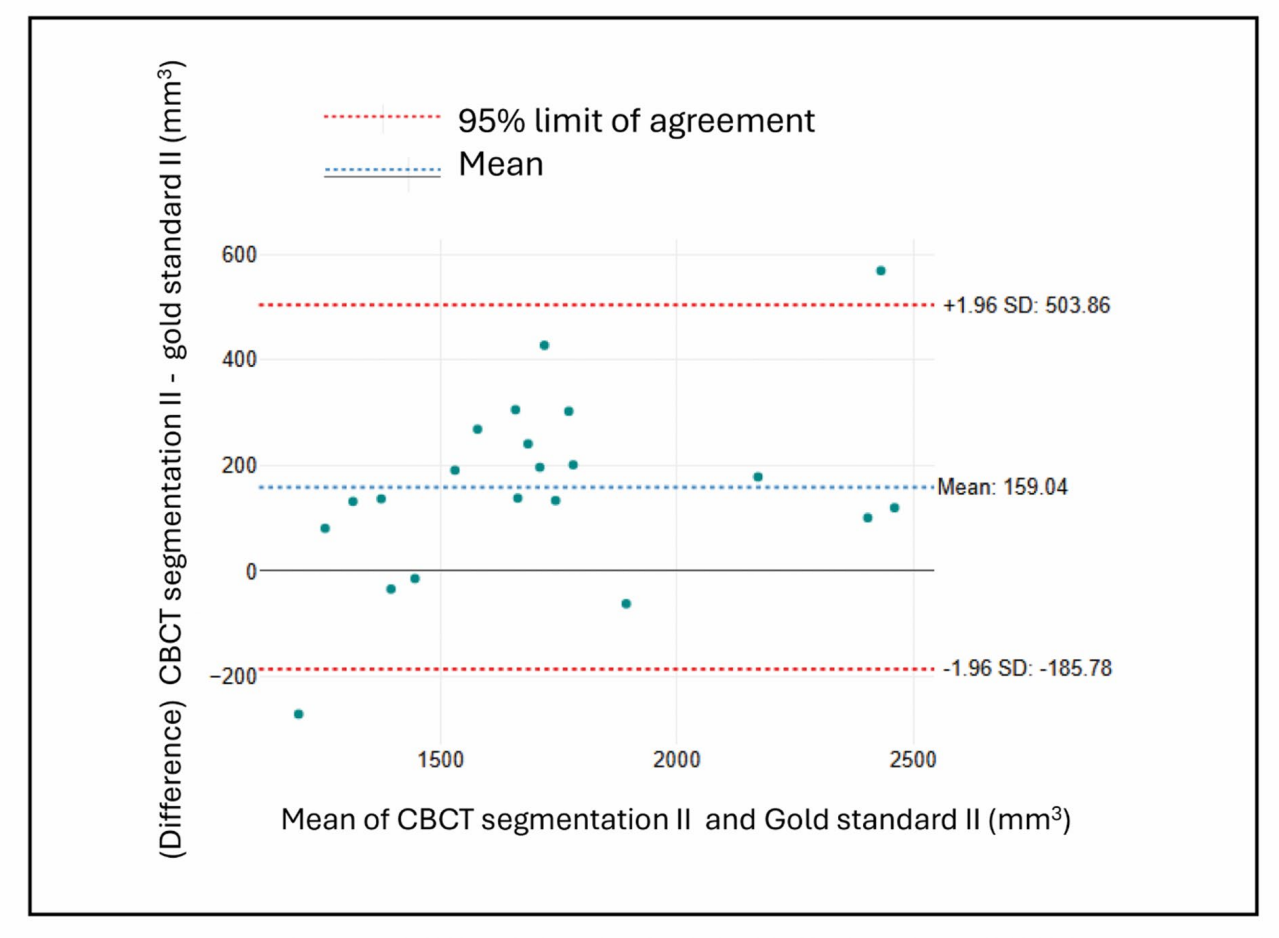
Bland-Altman plot comparing CBCT and gold standard measurements in group II.

For the CBCT segmentation methods, (Figs. 10 and 11) illustrate less favorable outcomes. Figure 9 (CBCT Segmentation vs. Gold Standard in group I defects) shows a slightly negative mean difference (-24.78 mm³), suggesting mild underestimation, accompanied by wide limits of agreement (approximately ± 504 mm³). Figure 10 (CBCT Segmentation vs. Gold Standard in group II defects) reveals a more pronounced positive bias (159.04 mm³), indicating consistent overestimation, with similarly broad variability (limits of approximately + 504 and − 186 mm³).

Overall, the IOS measurements exhibited smaller biases and narrower limits of agreement compared to CBCT segmentation, indicating that IOS provides more accurate and consistent volumetric assessments of bone defects.

## Discussion

CBCT is a 3D imaging modality widely used in the oral and maxillofacial region, particularly for diagnosing and planning the treatment of bone defects. Its ability to provide accurate volumetric data is crucial for evaluating treatment response, comparing different therapeutic approaches, and monitoring defect healing over time^8^. Our study aimed to evaluate and compare the accuracy and reliability of CBCT and IOS in measuring the volume of simulated bone defects.

In this study, bovine rib bone was selected as a replacement for human bone despite differences in bone density and mechanical properties. Nonetheless, the structural similarity between the cortical bone and bone marrow of bovine and human bones makes it a suitable alternativ^9^. Several prior investigations have demonstrated its utility in preclinical research. For instance, Schwarz et al.^10^ used bovine bone blocks to create standardized defect models for evaluating guided bone regeneration techniques. Nevins et al.^11^ employed them to assess the osseointegration potential of dental implants within the cortical bone.

In the present study, the accuracy of CBCT-based volumetric measurements was found to be strongly influenced by the integrity of the cortical walls surrounding the defect. For Group I defects, which involved perforation of only one cortical plate, no statistically significant difference was detected between CBCT-derived volumes and those obtained by the gold standard technique (*P* = 0.26). In contrast, Group II defects, characterized by perforation of both cortical plates, showed a statistically significant difference between CBCT segmentation and the gold standard (*P* = 0.001).

This clear distinction between the two groups highlights the critical role of cortical wall preservation in maintaining segmentation accuracy. The absence of structural boundaries eliminates the high-contrast interface that typically guides contour tracing during manual segmentation. As a result, the segmentation process may be prone to subjectivity and overestimation. This phenomenon was reflected in the positive bias (159.04 mm³) observed in Group II volumetric measurements compared with the gold standard. The overestimation tendency likely arises from the segmentation extending beyond the true defect limits.

Our study results are consistent with the observations of Vallaeys et al.^3^ who noted that the absence of cortical borders frequently leads to segmentation overshoot. Schulze et al.^12^ also emphasized that CBCT artifacts and reduced contrast resolution, especially in poorly defined regions, further compromise accurate delineation of defect margins. Likewise, Pauwels et al.^13^ attributed volumetric inaccuracies in such cases to partial volume effects and beam hardening artifacts, which become more pronounced when cortical boundaries are absent.

In contrast to our findings, El-Beblawy et al.^14^ and Agbaje et al.^15^ both reported significant discrepancies between CBCT-derived and gold standard volumes, even in less complex defect models. These differences may be explained by the segmentation method employed. Both studies used semiautomatic segmentation, which depends heavily on threshold settings and algorithmic boundary detection. Incorrect or generalized threshold selection may misrepresent the true extent of a defect, resulting in systematic under- or overestimation. In comparison, our manual segmentation approach, though more operator-dependent, allowed finer control in defining the margins of complex or irregular defects, particularly when one cortical wall was absent.

To our knowledge, this study is among the first to systematically investigate how the progressive loss of cortical walls affects the volumetric accuracy of CBCT-based measurements within a controlled experimental model. These results underline the importance of considering cortical wall integrity when interpreting CBCT volumetric data in both research and clinical contexts.

Regarding interexaminer reliability in our study, a good level of agreement was observed for manual segmentation using MIMICS software, with an ICC of (0.87). This reflects satisfactory consistency between examiners in delineating defect volumes. In comparison, El-Beblawy et al.^14^. reported a higher interexaminer agreement (ICC = 0.99) using the same software, suggesting near-perfect reproducibility. Similarly, Weissheimer et al.^16^ evaluated the accuracy of six different imaging software programs, including MIMICS. Their findings demonstrated high repeatability for the volumetric measurements, with an ICC of 0.94. It is important to note that both studies employed semi-automatic segmentation techniques, which are inherently less operator-dependent due to their reliance on algorithm-driven processing. Such methods reduce variability by limiting the influence of manual input, potentially explaining the higher interexaminer reliability reported in those studies compared to ours.

The integration of IOS into clinical and research workflows holds significant promise for radiation-free monitoring of bone healing and the development of digital twin models in reconstructive surgery. By providing highly accurate 3D surface data without ionizing radiation, IOS enables longitudinal assessment of bone defect healing, which is a major advantage for follow-up in implantology, regenerative procedures, and post-surgical evaluation^17–20^.

Despite the promising potential of IOS, the comparative evidence between IOS and CBCT in bone defect volumetry remains limited. Only a few investigations have explored this aspect. Kamburoğlu et a^5^. compared the accuracy of IOS and CBCT in detecting the volume of artificially created maxillary defects and reported that IOS-derived volumes were more accurate. Similarly Lindstrِm et al. [4] have assessed the accuracy of the volumetric measurements obtained by TRIOS IOS (3Shape TRIOS, 3Shape A/S, Copenhagen, Denmark) of simulated bone defects and suggested that the IOS volumes possessed high levels of accuracy. These results match the results of our study, which reported that IOS possessed higher accuracy than CBCT in measuring the volume of artificially created bone defects of both groups.

Moreover, a significant practical advantage for the IOS-based workflow is its dramatic reduction in analysis time. The CBCT-based volume analysis in our study required an average of 30 min per defect. In contrast, the IOS workflow required only 2 min per defect. This vast difference highlights the efficiency of IOS. In clinical settings where volumetric monitoring might be frequent, this time savings translates directly into reduced workload and faster feedback, complementing its superior accuracy in defect volume assessment.

The novelty of our study lies in its dual comparative approach, which evaluates the accuracy of both CBCT and IOS in quantifying the volume of two osteolytic defects exhibiting distinct morphological characteristics.

In conclusion, IOS demonstrated superior accuracy over CBCT segmentation in measuring the volume of artificially created bone defects. This was most evident when both cortical plates were absent, where IOS measurements showed no significant deviation from the gold standard volume (*P* = 0.88), whereas CBCT measurements showed a significant deviation (*P* = 0.001). Thus, IOS presents a reliable, radiation-free alternative for volumetric bone defect monitoring across varying degrees of cortical bone loss.

However, several limitations should be acknowledged. First, this study was conducted in vitro, and therefore, the results require validation in vivo. Clinical conditions present challenges that may impact the scanning process, such as the presence of saliva or blood, limited access to the area of interest, and patient movement during scanning. Second, although the Archimedes principle is considered a reliable volumetric measurement method, its accuracy is influenced by the choice of material, its ability to adequately fill and flow into the volume of interest, and its stability after removal. Finally, manual segmentation has several drawbacks. It is time-consuming, relies heavily on the skill of the operator, and can vary between different examiners.

Future studies should examine the accuracy of semi-automated CBCT segmentation, investigate the integration of deep learning algorithms for automation, and conduct comparative efficiency assessments between manual, semi-automatic, and fully automatic segmentation methods. Additionally, we recommend the use of IOS as a highly precise alternative to the Archimedes principle for volume measurement, particularly at the research level.

## Data Availability

All data included in this study are available from the corresponding author upon request.
